# Multi-joint isometric measurement for the evidence-based assessment of upper limb strength impairment in wheelchair athletes with different health conditions: a preliminary study

**DOI:** 10.5114/biolsport.2023.119286

**Published:** 2022-11-14

**Authors:** Marta Domínguez-Díez, Javier Raya-González, Jose L.L. Elvira, Raul Reina

**Affiliations:** 1 Faculty of Sport Sciences. Universidad Europea de Madrid, Madrid, Spain; 2 Faculty of Health Sciences, Universidad Isabel I, Burgos, Spain; 3 Sports Sciences Department, Miguel Hernández University, Elche, Spain

**Keywords:** Paralympic, Classification, Physical impairment, Wheelchair sports, Force

## Abstract

The present study presents a novel specific multi-joint isometric test to assess upper limb strength impairment for evidence-based classification in wheelchair sports. Sixteen wheelchair athletes participated in this study and were classified according to their type of physical impairment and health condition as follows: athletes with neurological impairment (ANI, n = 5) and athletes with impaired muscle power (IMP, n = 11). In addition, six non-disabled participants formed a control group (CG, n = 6). All the participants performed the isometric propulsion strength test (IPST), evaluating pushing and pulling actions, and two wheelchair performance tests. Excellent relative intra-session reliability scores were obtained for strength values for the ANI, IMP and CG groups (0.90 < ICC < 0.99) and absolute reproducibility showed acceptable scores of SEM (< 9.52%) for IPST pushing action. The ANI had significantly lower scores in strength and wheelchair performance than the IMP and the CG, while no differences were found between the IMP and the non-disabled participants. In addition, no correlations were found for wheelchair athletes between the isometric upper limb strength measure and wheelchair performance. Our findings suggest that the IPST is a valid test for strength measurement in upper limb impairment wheelchair athletes with different health conditions, which must be used in combination with a performance test to obtain a holistic assessment of this population.

## INTRODUCTION

Manual muscle testing has been widely used in classification in Paralympic sports to infer loss of strength by rating muscle resistance at different positions or ranges of movement [1]. However, this measurement is questioned due to the poor reliability as a result of the subjective assessment of muscle strength and the ordinal measures applied, which are also limited in defining their relationship with sporting performance [1–3]. For the purpose of inferring loss of muscle strength in Paralympic classification, isometric tests appear to have the most validity as they are (a) less training responsive; (b) instrumented, yielding a ratio-scale measure; (c) comprehensive, assessing all muscle actions of importance; and (d) parsimonious, assessing compound (or multi-joint) actions [2, 4]. In this regard, some authors have recently published key recommendations to develop isometric measures, suggesting that the most appropriate method to assess impaired strength in para-athletes should be multijoint and isometric tests performed at specific joint angles that facilitate maximum force production in a standardized and sport-specific position [2]. Previous research suggested that isometric strength measures are valid to produce clusters of athletes with similar levels of activity limitation in wheelchair racing [5] and Para-swimming [6] and show high reliability across various upper body strength tests in the non-disabled population [6, 7], providing methodological guidance for the development of evidence-based classification systems. Maximal peak force has been suggested as the key outcome variable in isometric protocols for classification purposes to assess strength, being claimed to be less training responsive [2]. Despite these promising findings, these methods may not be valid for all disabled subjects, so future research is imperative to develop and evaluate them on wheelchair athletes with different health conditions.

Wheelchair athletes with health conditions such as spinal cord injury (SCI), amputations or cerebral palsy (CP) could present different manifestations of impaired strength and, consequently, their activity limitation is determined by the muscles affected and the extent to which voluntary force production is reduced [5, 6]. Whereas athletes with amputations or SCI tend to present a loss of voluntary motor control below the level of the lesion affecting the trunk and lower limb structures, neurological disorders have inconsistent manifestations, affecting upper limb, trunk and lower limb control movement [8]. Athletes with neurological impairments resulting in ataxia, hypertonia or athetosis (e.g., CP) tend to present highly affected neuromuscular functions, producing a loss of voluntary motor control that varies considerably depending on the severity and the location of the impairment [9–11]. Specifically, the presence of spasticity, weakness and abnormal dynamic activation patterns in those para-athletes produces a global motor dysfunction, and it limits their capability to produce force during movement and motor performance [12], which affects the range of motion [13], the ability to rapidly generate force, and the ability to generate maximum force [14]. Considering the aforementioned literature, it seems pertinent to consider that athletes with CP will present less upper limb strength than healthy subjects, which could cause decreases in wheelchair performance (e.g., change of direction ability), although this relationship is still unknown.

In wheelchair court sports, the key determinants of mobility performance are the ability of the athlete to accelerate, sprint, brake and turn with the wheelchair [15, 16], so pushing and pulling actions are the main relevant movements. To ensure that an isometric test is valid, sport-specific and related to performance, the participants’ position during the test should be related as much as possible to the propulsion actions [2]. Isometric strength tests using multiple joints have been shown to have a stronger relationship with athletic performance [17–19], especially when the most specific positions are used and the muscles contributing to the force production are representative of the activity of interest. Upper limb maximal strength has been correlated with wheelchair propulsion acceleration and top speed in wheelchair rugby [20] and wheelchair racing [5] athletes, respectively. During wheelchair propulsion, wheelchair athletes are required to follow the path of the push rim, being part of a shoulder to hand closed chain [21, 22]. Shoulder flexion power has been reported as an essential contributor to manual wheelchair propulsion and starting push phase kinematics has been described with a flexion and adduction shoulder movement from an extended and abducted initial position [22–24]. Thus, the most parsimonious and sport-specific approach would be a single test, with each athlete positioned in his/her own wheelchair pushing maximally on the push rim, involving key pushing muscle groups [5].

The present study presents a novel specific isometric test to assess upper limb strength impairment for evidence-based classification in wheelchair sports. The aims of this study were (i) to determine the relative and absolute within-day reliability of a sport-specific isometric strength test to assess upper limb strength impairment for wheelchair athletes, (ii) to assess the capacity of a specific multijoint isometric test to determine upper limb strength impairment in wheelchair athletes with different health conditions and non-disabled participants, and (iii) to establish the strength of association between upper limb isometric strength and wheelchair performance.

## MATERIALS AND METHODS

### Experimental design

A cross-sectional design was used to assess the reliability of the isometric propulsion strength test (IPST) and to identify impaired upper limb muscle strength and its relationship with wheelchair propulsion performance. In a prior session the chief researcher visited the three participant clubs and explained the procedures and aim of the investigation to participants and coaches. Testing was conducted over a single session and was supervised by the research team consisting of experts in evidence-based classification sport science. Participants performed the IPST and two wheelchair performance tests – the wheelchair change of direction ability test (WCODA) and the linear wheelchair sprint test (LWST) – in a randomized order. A rest interval of 5 min was allowed between each test in order to reduce the fatigue effects. Prior to the assessment session, wheelchair athletes participated in two familiarization sessions, in which they performed all the tests, with corrections, after the pertinent explications. The experimental protocol was completed on a synthetic indoor court, and the wheelchair athletes used their specific sports equipment (e.g., gloves, strapping). Two wheelchairs (Quickie All Court, Sunrise Medical, Torrance, California) were used by the control group for testing, with the same strapping system as their counterparts. Before each testing session a standardized warm-up consisting of 5 min of self-paced, low-intensity wheelchair propulsion, dynamic stretching and six 5 m sprints was performed.

### Participants

Sixteen wheelchair athletes with different physical impairments and six non-disabled participants were recruited from three regional clubs of wheelchair basketball, wheelchair slalom and paratriathlon and were divided into two sub-groups according to the origin of their eligible impairment. Five athletes with spastic and mixed forms of CP (age = 32 ± 10 years; sitting height = 81 ± 6 cm; body mass: 70 ± 14 kg; body mass index = 32 ± 9 kg/m², training experience = 5.5 ± 3 years) composed the sub-group of athletes with neurological impairment (ANI), which presented as global impairment, including upper limbs, lower limbs and trunk. Participants with medical conditions such amputation (n = 3), incomplete lumbar SCI (n = 4) and spina bifida (n = 4) comprised the impaired muscle power sub-group (IMP) (age = 36 ± 11 years; sitting height = 88 ± 10 cm; body mass = 74 ± 19 kg; body mass index = 28 ± 4 kg/m^2^, training experience = 4.5 ± 3.2 years), with impairment below the injured area (upper limbs not affected). Non-disabled participants comprised the control group (CG) (age = 30 ± 4 years; sitting height = 84 ± 5 cm; body mass = 68 ± 7 kg; body mass index = 23 ± 2 kg/m²). Wheelchair athletes’ coaches and adapted physical education teachers formed the CG to ensure regular handling and manoeuvrability of the wheelchair. All participants signed an informed consent form after being informed about the aims and procedures of the experiment and participated voluntarily. The study was conducted according to the Declaration of Helsinki, and the protocol was fully approved by the ethics committee of the authors’ university.

### Procedures

**FIG. 1:**
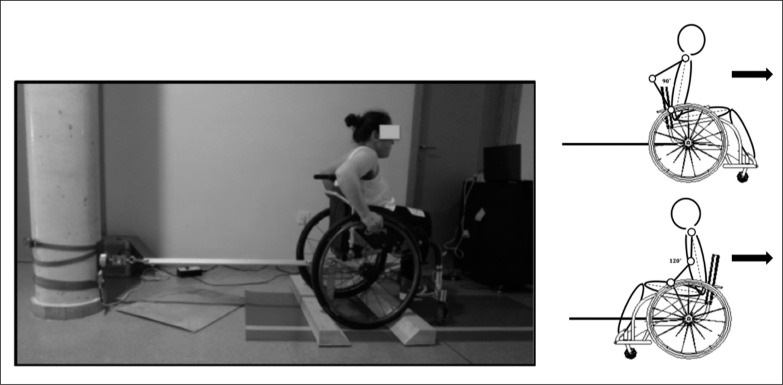
Schematic representation of the isometric propulsion strength test configuration for push and pull actions.

*Isometric propulsion strength test* (*IPST):* Participants were required to perform push and pull propulsion actions. A strain gauge load cell (Globus Iso Control, Codogné, Italy) was used to register isometric wheelchair propulsion forces. The load cell was instrumented on the athletes’ wheelchairs, attached and centred on webbing above the wheels’ axes, and fixed to a rigid structure aligned horizontally through a rigid bar (94 × 2.5 × 2.5 cm). The wheelchair was blocked up and a non-slip surface was used to prevent the wheels from turning. Elbow flexion angle was standardized at 90º for the push action and 120º for the pull action for all the participants [25, 26] (Figure 1). Trunk flexion was standardized within 70–80º to the horizontal for push and pull actions and corrected before each repetition. A goniometer (Lafayette model 01135) was used to check the standard elbow and trunk positions and a real-time video feedback system was used to ensure the correct position during executions [5].

Once positioned, participants performed six maximum, voluntary contractions (firstly three push actions and then three pull actions) of 5 s duration each, separated by 2 min for resting. Wheelchair positions were turned 180º during the rest period to perform push and pull actions. Prior to the initiation of the test series researchers ensured that participants understood the test procedure and participants performed two submaximal practice trials. All participants were monitored by the same tester and instructed to gradually build towards maximal force over the first 2 s and to maintain that force for the remaining 3 s, based on previous recommendations [5]. The instantaneous force was displayed in real time to ensure that the trials were valid and visual feedback of the force-time curve was also provided to participants. The horizontal force applied on the wheelchair was registered with the load cell and digitalized by a Data Acquisition Toolbox (DAQ, National Instruments, USB-1208FS) into a 5 V signal. The signal was sampled at 500 Hz by the software Tracer Daq (Measurement Computing Corporation, Norton, MA, USA) to generate a visual feedback display during the test. Force data analysis was conducted with LabVIEW (v.2010, National Instruments, Austin, USA) and low-pass filtered at 50 Hz and maximal resultant peak force (F_peak_) was analysed. The best of the trials in each action was used for the statistical analysis considering the maximal force executed [5, 6].

**FIG. 2:**
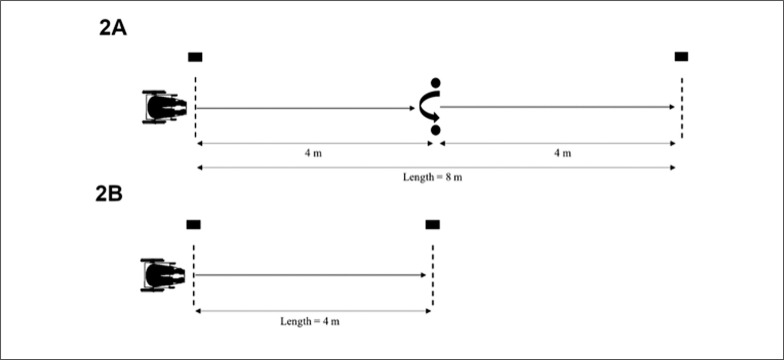
Schematic representation of the wheelchair change of direction ability test (2A) and the linear wheelchair sprint test (2B).

*Wheelchair change of direction ability (WCODA) test:* The participants propelled themselves forward 8 m in a straight line. At 4 m, participants crossed back between two wood cylinders (40 cm height, 10 cm diameter, 1.75 kg weight), turning 180º, which required a pulling action. This test was adapted from Altmann et al. [27] to assess acceleration ability in a linear sprint incorporating a change of direction. These equipment and distances are specific to the wheelchair slalom para-sport. Participants completed three trials with 2 min of recovery between trials. Time measurement was registered by timing gates (Globus, Codogné, Italy) placed 1 m above the ground over the start and finish lines. This height was established as the mean shoulder height from the ground of the participants seated in their own wheelchairs. The best score was used for further statistical analysis (Figure 2A).

*Linear wheelchair sprint test (LWST)*: The participants performed three maximal wheelchair sprint tests of 4 m distance, with a rest period of 2 min between trials [27]. Timing gates (Globus, Codogné, Italy) were positioned at the start and finish lines 1 m above the ground. Participants were instructed to “cross the 4 m line at maximal speed.” The best score was used for further statistical analysis (Figure 2B).

### Statistical analyses

Data are presented as mean ± standard deviations (SD). The normal distribution of the results of the variables applied was tested using the Kolmogorov-Smirnov test, and statistical parametric techniques were carried out. Relative and absolute reliability among trials in each test was assessed using intra-class correlations (ICCs) and standard error of measurement (SEM) respectively [28]. Intra-session reliability was calculated as the immediate test-retest reliability related to the random variability of the measurement per se that could be subject to the inaccuracy of the measurement or athlete’s performance variations. ICC values were calculated and categorized as excellent (0.90–1.00), high (0.70–0.89), moderate (0.50–0.69), or low (< 0.50) [29]. The SEM was calculated using the following formula: SEM = SD and expressed as a percentage of the mean scores (SEM%) considering values lower than 10% as acceptable [30].

A one-way analysis of variance (ANOVA) with a least significant difference post hoc comparison (Bonferroni correction) was used to examine isometric strength push and pull actions and wheelchair performance mean differences among groups (i.e., ANI, IMP and CG). Practical significance was assessed by calculating Cohen’s effect size (ES) [31]. ES of above 0.8, between 0.8 and 0.5, between 0.5 and 0.2, and lower than 0.2 were considered as large, moderate, small, and trivial, respectively. Paired comparisons among groups for each variable were expressed using mean differences, calculated as: mean difference (%) ((mean 1 – mean 2)/mean 2) × 100. Pearson’s product-moment correlation coefficient (r) with a 90% confidence interval (CI) was used to examine the relationship between isometric strength test and wheelchair performance for each group. The following scale of magnitudes was used to interpret the correlation coefficients: < 0.1, trivial; 0.1–0.3, small; 0.3–0.5, moderate; 0.5–0.7, large; 0.7–0.9, very large; and > 0.9, nearly perfect [30]. If the 90% confidence limits (CLs) overlapped small positive and negative values, the magnitude was deemed unclear; otherwise, the magnitude was deemed to be the observed magnitude [32]. Data analysis was carried out using IBM SPSS Statistics for Windows, version 25.0 (IBM Corp., Armonk, NY, USA). Statistical significance was set at p < 0.05.

## RESULTS

**FIG. 3:**
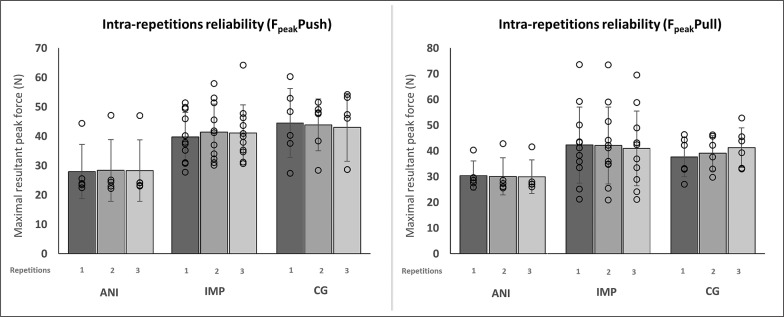
Intra-repetition reliability related to force values (push and pull) for each group. ANI = athletes with neurological impairment; IMP = athletes with impaired muscle power; CG = control group.

Within-session reliability for each player was evaluated among the three trials performed. Excellent relative intra-session reliability scores were found for F_peak_ values for the ANI, IMP and CG groups (0.90 < ICC < 0.99) and absolute reproducibility showed acceptable scores of SEM (< 9.52%) for the IPST pushing action. On the other hand, IPST pull action showed higher values of SEM for the ANI (SEM = 13.50%) and IMP (SEM = 11.62%) while CG absolute reliability was SEM = 5.56%. The intra-session reliability of the two performance tests shows excellent-high reliability (0.80 < ICC < 0.99) for all participants’ scores and good SEM values (< 5.88%). Intrarepetition reliability related to force values (push and pull) for each group is presented in Figure 3.

Results observed for the isometric propulsion strength and wheelchair performance in each group are presented in Table 1. The ANI obtained the worst values for the F_peak_Push (p < 0.05; ES = -1.47/-1.67) and LWST (p < 0.01; ES = 1.40–1.91) in comparison with IMP and CG groups. Additionally, ANI showed a worse performance for WCODA (p < 0.01; ES = 1.79) compared with IMP. No significant differences (p < 0.05) were found between IMP and CG.

**TABLE 1:** Differences in the isometric propulsion strength and wheelchair performance mean values among groups.

	Mean ± SD [confidence intervals (95%)]			Mean difference (%); ES, interpretation		
	ANI (n = 5)	IMP (n = 11)	CG (n = 6)	ANI-IMP	ANI-CG	IMP-CG
F_peak_Push (N)	28.6 ± 10.4	43.6 ± 10.1	46.9 ± 11.5	52.4; -1.7, large*	64.3; -1.7, large*	7.8; -0.3, small
	[19.3–37.9]	[37.6–49.6]	[37.7–56.1]			

F_peak_Pull (N)	32.9 ± 11.2	44.7 ± 16.5	41.7 ± 7.8	35.9; -0.8, moderate	26.8; -0.9, large	6.8; 0.2, small
	[22.9–42.9]	[35.0–54.4]	[35.5–47.9]			
WCODA (s)	10.5 ± 3.4	6.5 ± 1.5	7.9 ± 0.9	37.4; 1.8, large**	24.4; 1.1, large	20.7; -1.0, large
	[7.5–13.5]	[5.6–7.4]	[7.2–8.6]			

LWST (s)	3.9 ± 1.5	2.4 ± 0.3	2.6 ± 0.2	40.9; 1.9, large**	35.7; 1.4, large*	8.9; -0.7, moderate
	[3.0 to 4.8]	[2.2–2.6]	[2.4–2.8]			

Abbreviations: SD = standard deviation; ES = Effect size; ANI = athletes with neurological impairment; IMP = athletes with impaired muscle power; CG = control group; F_peak_Push = maximal horizontal peak force in push action; F_peak_Pull = maximal horizontal peak force in pull action; WCODA = wheelchair change of direction ability test; LWST = linear wheelchair sprint test.

* Significant level set at p < 0.05;

** Significant level set at p < 0.01.

The relationships among IPST and the wheelchair performance tests for the ANI, the IMP, and the CG are presented in Table 2. For the ANI and the IMP, these relationships were unclear when compared to the isometric propulsion strength and wheelchair performance tests. However, in the CG participants who obtained higher values of isometric strength in the push condition showed better performance in the LWST (r = 0.89; ± 0.7, very large, p < 0.05). Moreover, the two IPSTs correlated for the ANI and the CG (r = 0.94–0.98; ± 0.32–0.48, near perfect, p < 0.05), whereas the two performance tests correlated (r = 0.84–0.99; ± 0.14–0.78, near perfect-very large, p < 0.05–0.01) for all the groups.

**TABLE 2:** Relationships (r) with a 90% confidence interval (CI) between the players’ isometric propulsion strength test and wheelchair performance test in each age group.

		F_peak_Push	F_peak_Pull	WCODA
**ANI**		–		
**IMP**	**F_peak_Pull**	–		
**CG**		–		

**ANI**		0.98 ± 0.32 NP**	–	
**IMP**	**F_peak_Pull**	0.13 ± 1.17 ?	–	
**CG**		94 ± 0.48 NP **	–	

**ANI**		-0.42 ± 1.69 ?	-0.43 ± 1.68 ?	–
**IMP**	**WCODA**	0.25 ± 1.11 ?	-0.07 ± 1.19 ?	–
**CG**		0.64 ± 1.30 ?	0.65 ± 1.30 ?	–

**ANI**		-0.4 ± 1.65 ?	-0.47 ± 1.65 ?	0.99 ± 0.14 NP**
**IMP**	**LWST**	0.40 ± 1.06 ?	0.16 ± 1.18 ?	0.84 ± 0.47 VL**
**CG**		0.89 ± 0.7 VL*	0.58 ± 1.06 ?	0.87 ± 0.78 VL*

Abbreviations: ANI = athletes with neurological impairment; IMP = athletes with impaired muscle power; CG = control group; F_peak_Push: maximal horizontal peak force in push action; F_peak_Pull: maximal horizontal peak force in pull action; WCODA: wheelchair change of direction ability test; LWST: linear wheelchair sprint test. Correlation magnitude: ?, unclear; S, small; M: moderate; L, large; VL, very large; NP, near perfect.

* Significant level set at p < 0.05;

** Significant level set at p < 0.01.

## DISCUSSION

The aim of this study was to propose a specific test to evaluate upper limb isometric strength in a multi-joint propulsion position to identify upper limb strength impairment in wheelchair athletes, while also establishing the strength of the association between these measures and wheelchair propulsion performance. This is the first study that correlates the performance regarding upper limb isometric strength in a multi-joint propulsion position with functional tests (i.e., WCODA and LWST) in wheelchair athletes. The main results of this study report good relative and absolute intra-session reliability of the proposed measures, being able to discriminate between wheelchair athletes with and without altered upper limb muscle force production. In addition, higher values of isometric strength in the push condition were related to better performance in the LWST for the non-disabled participants.

Although previous studies have shown excellent reproductivity of the isometric upper limb test, assessing F_peak_ in wheelchair athletes [33] and the non-disabled population [7], the feasibility and reliability of those measurements have not been studied for specific wheelchair multi-joint pushing and pulling positions. The analysis of the reproducibility of F_peak_ in the IPST showed excellent relative values (ICC > 0.90) for wheelchair athletes with different health conditions (ANI and IMP) and non-disabled participants (CG). Additionally, acceptable scores of absolute reliability (SEM) were obtained for the pushing action, in spite of the higher SEM values for the pulling action for the ANI (SEM = 13.50%) and IMP (SEM = 11.62%), when compared to the pushing condition. Although these results reflect the intra-session consistency of the measure for all participants and could be considered to assess upper limb strength impairment in wheelchair athletes with different physical impairments, future studies are necessary to verify the test-retest reliability.

Previous researchers agree that test batteries developed for the purposes of evidence-based classification should favour multi-joint actions wherever possible and a sport-specific approach to assess upper limb isometric strength in wheelchair athletes [5, 33]. Our results showed that ANI produced less isometric force (F_peak_) during all test conditions compared to the IMP and CG participants, being significant when comparing results from the push test, and suggesting more upper limb strength impairment in those athletes with impairments of neurological nature. These results are in line with previous investigations of propulsion mechanics, which reported that individuals with upper-limb impairment (i.e., CP and multiple sclerosis) showed a lower power output along with a lower resultant force, propulsive moment (M_z_), and overall resultant moment on the hand rim during propulsion than users without upper limb impairment (i.e., SB, amputations, thoracic and lumbar SCI) [13, 34]. Conversely, the results obtained by Hogarth et al. [6] show similar upper limb strength values between para swimmers with hypertonia (e.g., CP) and impaired muscle power (e.g., incomplete and complete SCI, SB and polio). These differences could be explained by the range of medical health conditions of the impaired muscle power group including athletes presenting a different distribution of strength impairment across their body structures.

The spastic form of CP and other related neurological conditions present neuromuscular disorders that affect upper and lower extremities involving global body muscle weakness, muscle shortening, and diminished selective motor control [35]. In addition, neural (e.g., motor unit recruitment) and muscular factors (e.g., morphology of muscle fibres) reduce voluntary muscle activation, and consequently a lower muscle force is produced [35, 36]. Thus, athletes with physical impairment such as hypertonia could present an inconsistent strength impairment distribution along all of the muscle structures required for optimal wheelchair propulsion, these being the shoulder flexors/adductors, the elbow extensors and wrist pronators [22]. Additionally, they could present motor coordination impairment and diminished range of motion that could affect the resultant force applied on the hand rim. It is pertinent to highlight that no differences were found between the IMP and the CG for the IPST, probably due to the injury level and health condition of wheelchair athletes from the IMP group, mainly involving athletes presenting trunk and lower limb strength impairment. These results are in line with those observed by Kotajarvi, Basford, & An [37], who did not find significant differences in maximum isometric shoulder strength when comparing participants with paraplegia with the able-bodied controls.

A key step towards evidence-based classification systems in parasport is developing valid tests of impairment and establishing their relationship with sports performance [38]. The results obtained in the current study show unclear correlations between IPST and the two performance tests for both sub-groups with physical impairments, which do not support previous findings. Regarding this, Connick et al. [5] reported that a multi-joint (combining arm push with trunk flexion) strength measure significantly correlated with a 15 m wheelchair propulsion sprint test. These results could be due to the fact that these authors conducted the correlation test using the overall sample. On the other hand, Mason et al. [33] reported that six upper body isometric strength tests correlated with a 2 m and 10 m sprint, although the sample (i.e., athletes without trunk function) and the used test (i.e., single-joint actions) were different from those in our study. Additionally, our results show a significant correlation between isometric strength in the push condition and wheelchair sprint in 4 m for the CG participants. An explanation for the above observation is that the type of physical impairment and health condition influences the association between the strength test and wheelchair performance. These results could be explained by the fact that the strength test might be associated with the level of spasticity that affects athletes with CP and may be collinear with other physical impairments such as motor coordination [39], whereas in athletes with SCI or SB trunk function impairment should be considered [40].

The main limitation of this study was the sample size in regard to the number of players of each group. A larger sample should be analysed, including more wheelchair athletes with a broader range of underlying health conditions leading to upper limb strength impairment. Also, physical activity between tests was not recorded, which could have influenced the obtained results. Likewise, for further investigation, it would be useful to assess the test-retest reliability of the multi-joint upper limb strength measure. To ensure greater reproducibility of the data, focusing a familiarization period could be employed for impaired participants for the IPST to ensure that variability is not a result of the nature of the impairment. Finally, while the isometric test measures have been correlated with wheelchair propulsion performance for the non-disabled participants, only total time was assessed. Other performance variables, such as maximal velocity or acceleration in the first propulsion, could be appropriate to analyse in studying wheelchair sports.

## CONCLUSIONS

In summary, the multi-joint isometric upper limb strength test presented in this study (IPST) show acceptable intra-session reliability and was found to differ between wheelchair athletes with upper limb impairment caused by neurological conditions and wheelchair athletes without upper limb impairment and non-disabled participants. This suggests that the IPST could be considered to provide a useful benchmark to evaluate upper limb impairment in wheelchair athletes with different health conditions, especially in those athletes who present inconsistent strength impairment distribution. The absence of correlations between strength measures and wheelchair sprint in wheelchair athletes questions the utility of the strength measures for the performance evaluation with this population. Finally, the results obtained could contribute to evidence-based methods of classification for wheelchair athletes with upper limb strength impairments, although further studies on this topic are necessary.
